# Cross-reactivity of commercially available anti-human monoclonal antibodies with canine cytokines: establishment of a reliable panel to detect the functional profile of peripheral blood lymphocytes by intracytoplasmic staining

**DOI:** 10.1186/s13028-015-0142-y

**Published:** 2015-09-11

**Authors:** Marcela L. Moreira, Elaine M. S. Dorneles, Rodrigo P. Soares, Camila P. Magalhães, Christiane Costa-Pereira, Andrey P. Lage, Andréa Teixeira-Carvalho, Olindo A. Martins-Filho, Márcio S. S. Araújo

**Affiliations:** Laboratório de Biomarcadores de Diagnóstico e Monitoração, Centro de Pesquisas René Rachou/FIOCRUZ-MG, Belo Horizonte, Minas Gerais Brazil; Laboratório de Bacteriologia Aplicada, Escola de Veterinária, Universidade Federal de Minas Gerais, Avenida Antônio Carlos 6627, Pampulha, Belo Horizonte, MG 31270-901 Brazil

**Keywords:** Cytokines, Canine, Cross-reactivity, Flow cytometry

## Abstract

**Background:**

The process for obtaining monoclonal antibodies against a specific antigen is very laborious, involves sophisticated technologies and it is not available in most research laboratories. Considering that most cytokines remain partially conserved among species during evolution, the search for antibody cross-reactivity is an important strategy for immunological studies in veterinary medicine. In this context, the amino acid sequence from human and canine cytokines have demonstrated 49–96 % homology, suggesting high probability of cross-reactivity amongst monoclonal antibodies. For this, 17 commercially available anti-human monoclonal antibodies [IL-1α, IL-1β, IL-2, IL-4, IL-5, IL-6, IL-8 (#1, #2), IL-10, IL-12, IL-13, IL-17A, IFN-γ (#1, #2), TNF-α (#1, #2) and TGF-β], were evaluated in vitro for intracellular cytokine detection in a stimulated canine blood culture by flow cytometry and confocal microscopy. Lymphocytes from peripheral blood of healthy and two unhealthy dogs were analyzed.

**Results:**

Eleven anti-human mAbs [IL-1α, IL-4, IL-5, IL-6, IL-8 (#1, #2), IL-12, IL-17A, TNF-α (#1, #2) and TGF-β] cross-reacted against canine intracellular cytokines. The specificity of the assays was not affected after Fc-blocking. Three anti-human cytokine mAbs [IL-4, IL-8 (#2) and TGF-β] when evaluated by confocal microscopy also cross-reacted with intracellular canine cytokines. The identification of human mAbs that cross-reacted with canine cytokines may support their use as immunological biomarkers in veterinary medicine studies.

**Conclusion:**

The identification of these 11 anti-human cytokine mAbs that cross-reacted with canine cytokines will be useful immunological biomarkers for pathological conditions by flow cytometry and fluorescence microscopy in dogs.

## Background

Cytokines are soluble proteins secreted by different cell subtypes including antigen-presenting cells (APC), endothelial and epithelial cells, bone marrow stromal cells, fibroblasts, keratinocytes, platelets and both lymphoid and non-lymphoid cells. They are involved in a wide range of interactions such as the development of cellular and humoral immune responses, induction of inflammatory responses, regulation of hematopoiesis, control of proliferation/differentiation and cell migration [1]. Some cytokines [such as interleukin (IL) 6] circulate in picomolar concentrations and may increase by 1000 times during infection or trauma [2]. The synthesis of appropriate amounts of tumor necrosis factor (TNF-α), IL-1 and IL-6 is clearly beneficial in response to infection, but higher levels may be relevant for immunopathological processes [3–6]. Cytokine detection during the immune response can be useful prognostic markers in several diseases and also provide assessment of vaccine efficacy [7–9].

Animal models are widely used in biological research. Dogs, for example, are excellent models for studies of immunosuppression and are frequently used for transplants and they are also important as reservoir of visceral leishmaniasis. However, differently from humans and mice, dog studies are hindered due to a more restricted repertoire of commercially available monoclonal antibodies (mAb) against cytokines [10]. Quantification of cytokines could be a useful tool for diagnosis and understanding inflammatory conditions of domestic animals [11]. Thus, there is great demand for reactive antibodies against canine molecules, especially anti-cytokines.

As the process for development of monoclonal antibodies is laborious, costly, requires sophisticated technologies and is not available in most research laboratories, studies regarding cross-reactivity of antibodies against different species of cytokines are needed. Several studies have been conducted to identify the existence of cross-reactivity of surface molecules mAbs in animals [10, 12–15]. In 1993, the First International Canine Leukocyte Antigen Workshop—CLAW was conducted with the aim of identifying antigens of canine leukocytes and monoclonal antibodies. Those recognized homologous antigens were classified by analogy according to the nomenclature “cluster of differentiation” (CD) in human and murine [16]. Since then, cross-reactions between molecules expressed by human and animal lymphocytes have been reported [17–20]. This phenomenon is expected when the amino acid sequence homology among cytokines from different species is at least 60 % [21]. Comparative studies of reactivity of mAbs with cells from different species have shown that each antibody can recognize different epitopes [12]. Kwong et al. [22] produced and tested two bovine monoclonal antibodies against ovine TNF-α and found one with satisfactory reactivity. Schuberth et al. [18] tested cross-reactivity of 164 mAbs for pigs against canine leukocytes, and obtained 11 % of reactivity. Those data indicated that the recognition of conserved epitopes in evolutionarily distant species such as dogs and pigs was very low. Pedersen et al. [23] showed the existence of cross-reactivity of mAbs against sheep, cattle and human cytokines to different species and found cross-reactivity for four antibodies (IL-4, IL-8, IFN-γ and TNF-α) and Dorneles et al. [20] demonstrated that anti-human IL-1-α, IL-6, IL-8, IL-17A and IL-10 mAbs cross-react with cattle, goat and sheep cytokines. Overall, data on cross-reactivity among mAbs against human cytokines and domestic animals are scarce, mainly when dog cytokines are concerned. Therefore, the aim of this study was to screen a panel of 17 mAbs against human cytokines for cross-reactivity against canine cytokines. Furthermore, we have also performed additional analyzes to test the applicability of these antibodies during immunopathological disorders in dogs.

## Methods

### Monoclonal antibodies (mAbs)

Seventeen commercially available human mAbs against cytokines were used. All were conjugated with phycoerythrin fluorochrome (PE). Information on manufacturer, target species, host and catalog numbers are provided in the Table 1. Two mAbs against bovine cytokines (Serotec, Kidlington, UK) known to recognize canine cytokines were used as controls (Table 1). Isotypic controls were also included as provided in Table 1.

**Table 1:** Monoclonal antibodies tested

Antibody	Target	Host	Isotype	Clone	Manufacturer	Catalog	Reactivity
Anti-IL-1α	Human	Mouse	IgG1	3643B314	BD	554561	+
Anti-IL-1β	Human	Mouse	IgG1	AS10	BD	340516	–
Anti-IL-2	Human	Mouse	IgG1	5344.111	BD	340450	–
Anti-IL-4	Human	Mouse	IgG1	8F-12	IQProducts	IQP162R	+
Anti-IL-5	Human	Rat	IgG2	JES139D10	BD	559332	+
Anti-IL-6	Human	Rat	IgG1	MQ2-13A5	BD	554545	+
Anti-IL-8 (#1)	Human	Mouse	IgG2	G265-8	BD	554720	+
Anti-IL-8 (#2)	Human	Mouse	IgG1	E8N1	Biolegend	511408	+
Anti-IL-10	Human	Rat	IgG1	JES3-16E3	BD	554467	–
Anti-IL-12	Human	Mouse	IgG1	C11.5	BD	554575	+
Anti-IL-13	Human	Rat	IgG1	JES10-5A2	BD	559328	–
Anti-IL-17A	Human	Mouse	IgG1	eBio64DEC17b	eBioscience	12-7179-73	+
Anti-IFN-γ(#1)	Human	Mouse	IgG1	B27	BD	559327	–
Anti-IFN-γ(#2)	Human	Mouse	IgG1	45-15	IQProducts	IQP160r	–
Anti-TNF-α(#1)	Human	Mouse	IgG1	B-C7	IQProducts	IQP163R	+
Anti-TNF-α(#2)	Human	Mouse	IgG1	mab11	BD	559321	+
Anti-TGF-β	Human	Mouse	IgG1	TB21	IQProducts	IQP169R	+
Isotype control	–	Mouse	IgG1	–	BD	555748	–
Isotype control	–	Mouse	IgG2	–	BD	556437	–
Isotype control	–	Rat	IgG1	–	BD	554685	–
Isotype control	–	Rat	IgG2	–	BD	557229	–
Anti-IL-4	Bovine	Mouse	IgG2a	CC303	Serotec	mca1820pe	+
Anti-IFN-γ	Bovine	Mouse	IgG1	CC302	Serotec	mca1783pe	+

### Animals and controls

Forty-seven Mongrel dogs (*Canis lupus familiaris*) included in this study were selected in veterinary clinics in Belo Horizonte, Minas Gerais, Brazil (45 healthy dogs) and at the Hospital Veterinário—Escola de Veterinária—Universidade Federal de Minas Gerais HV—EV—UFMG (two diseased dogs). In this cross-reactivity study, cells collected from minimum 8 until 45 healthy dogs were evaluated. Further, one dog with peritonitis and another with dermatitis were included to evaluate the applicability of a selected set of anti-cytokine mAbs to characterize the immunological status under pathological disorders. Blood samples from four human volunteers were used as controls. Five ml of canine and human peripheral blood were collected in heparinized tubes. This study was approved by the Ethical Committee for the use of Experimental Animals (CEUA—P-71/11-3) from the Oswaldo Cruz Foundation, Brazil.

### In vitro culture

Short-term whole blood culture was performed according to Teixeira-Carvalho et al. [24] with modifications. Briefly, 1 ml of blood samples were cultured in 1 ml of RPMI (Invitrogen, Carlsbad, CA, USA) (37 °C, 5 % CO_2_, 4 h). Culture controls were incubated only in the presence of leukocytes plus RPMI. Stimulated cultures were incubated in the presence of leukocytes plus phorbol 12-myristate 13-acetate (PMA, 25 ng/ml, Sigma, St. Louis, MO, USA), ionomycin (ION, 1 μg/mL, Sigma, St Louis, MO, USA), and 1 μg/ml of lipopolysaccharide (LPS, Sigma, St Louis, MO, USA) (PMA + IONO + LPS). Also, Brefeldin A (BFA, 10 μg/ml, Sigma, St Louis, MO, USA) was added for all blood cultures to block cytokine transport. Following incubation, the cultures were treated with 2 mM ethylenediamine tetra acetic acid (EDTA) (Sigma, St Louis, MO, USA) and maintained at room temperature for 15 min.

### Intracellular cytokine immunostaining

#### Flow cytometric immunophenotyping of intracellular cytokines

Following the short-term in vitro stimulation, erythrocytes were lysed using 6 ml of FACS Lysing Solution (Becton–Dickinson, San Jose, CA, USA) under vortexing. After 5 min, the suspension was centrifuged (600*×g*, 7 min, room temperature). Then, the samples were incubated with FACS Permeabilizing Solution (0.015 M phosphate buffered saline—PBS, supplemented with 0.5 % bovine serum albumin, 0.5 % of saponin (Sigma, St Louis, MO, USA), and 0.1 % sodium azide, Becton–Dickinson) for 7 min. The suspension was centrifuged again (600×*g*, 7 min, room temperature). Then, cells were resuspended in 1 ml of FACS Permeabilizing Solution. During standardization procedure, 50 μl of suspension were incubated with several dilutions of PE-labeled anti-human cytokine mAbs (1:10, 1:25, 1:50 and 1:100), yielding a final concentration 0.1–1 μg/ml. According to the titration curve, the final concentration of 0.5 μg/ml for all tested antibodies (1:50 dilution of commercial available mAbs). The cells were incubated in the dark for 30 min at room temperature. After intracytoplasmic cytokine staining, cells were washed once (400×*g*, 10 min, 4 °C) with FACS buffer, fixed with 200 μl of FACS FIX Solution (10 g/l of paraformaldehyde, 10.2 g/l sodium cacodilate, 6.63 g/l sodium chloride, pH 7.2), and stored at 4 °C prior to flow cytometry acquisition and analysis. In all experiments, internal controls including unstained cells along with isotypic matching mAbs and reference cross-reactivity reagents (anti-bovine mAbs—IFN-g and IL-4 from Serotec, Kidlington, UK) were added in order to validate the cross-reactivity patterns.

The evaluation of cross-reactivity was conducted in three steps. First, the reactivity pattern of human mAbs was addressed by comparing the labeling profile of canine lymphocytes with that observed with human lymphocytes. Second, the cross-reactivity was confirmed by using internal controls (isotypic, blocking and reference cross-reactive mAbs). Blocking was carried out by parallel incubation of cells with 2 % of host serum (mouse or rat) during the incubation with anti-human cytokine mAbs. Third, the cross-reactivity pattern was further validated by comparing the frequency of cytokine positive cells observed in healthy and unhealthy dogs.

#### Flow cytometry acquisition and analysis

**Fig. 1:**
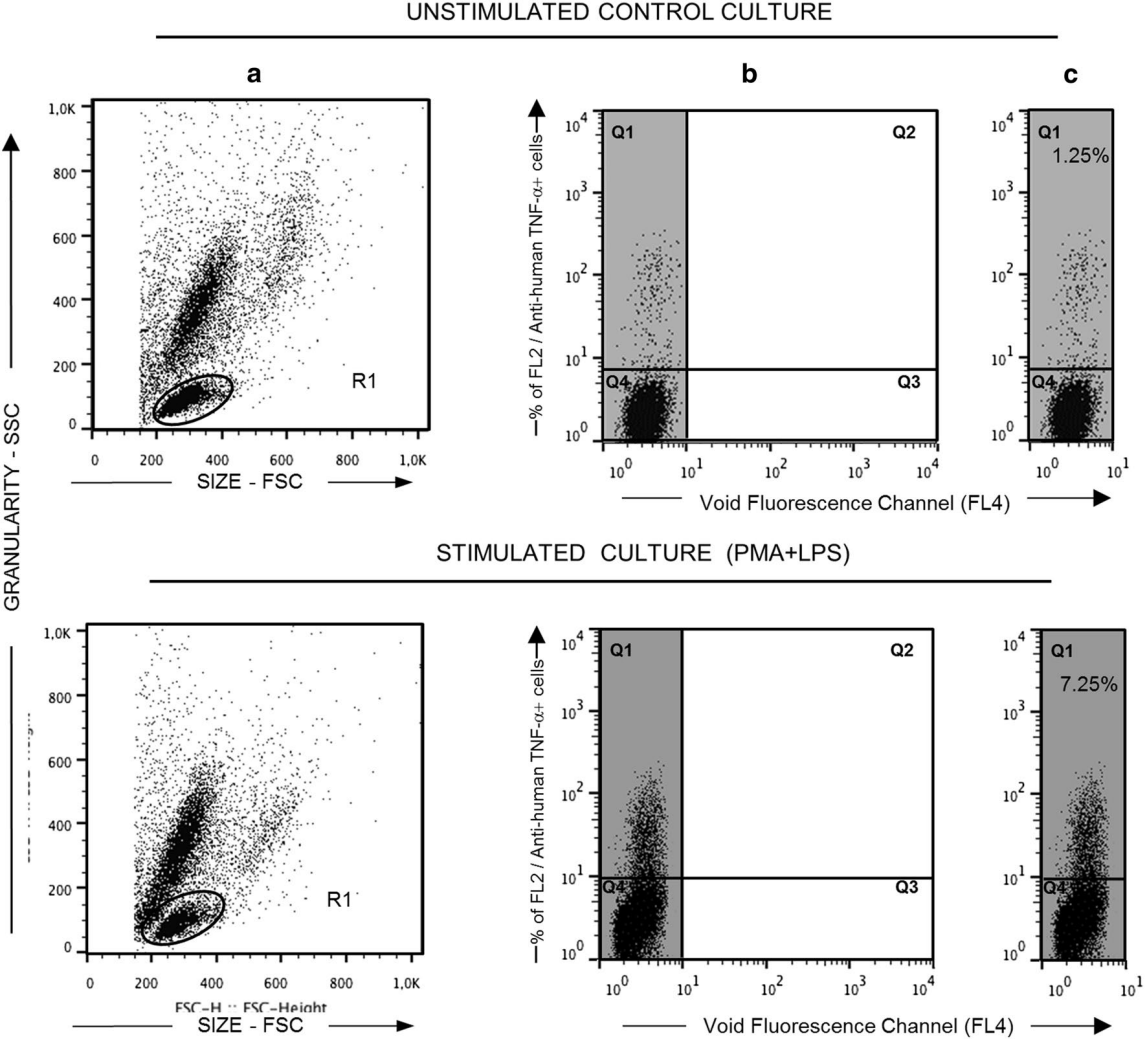
Gating strategy to quantify cytokine^+^ canine lymphocytes following short-term whole blood cultures in vitro. *Upper panels* (controls) and* lower panels* (stimulated with PMA + LPS). **a** A plot of spot size distribution (FSC) versus granularity (SSC) used for the selection of the lymphocyte population of interest—with the (R1) phenotype FSC × SSC. **b** *Dot plot* distribution of cytokine^+^ cells built by bi-dimensional graphs of empty channel (FL4 fluorescence) versus PE-emission channel (FL2) were used to quantify the percentage of cytokine^+^ lymphocytes within R1. The *left part* of the *graph* (Quadrants Q1 and Q4, highlighted by *gray background*) was used to compose the figures for subsequent analyses. **c** In the Q1 quadrant are shown the percentage (%) of cytokine^+^ lymphocytes using TNF-α as example

**Fig. 2:**
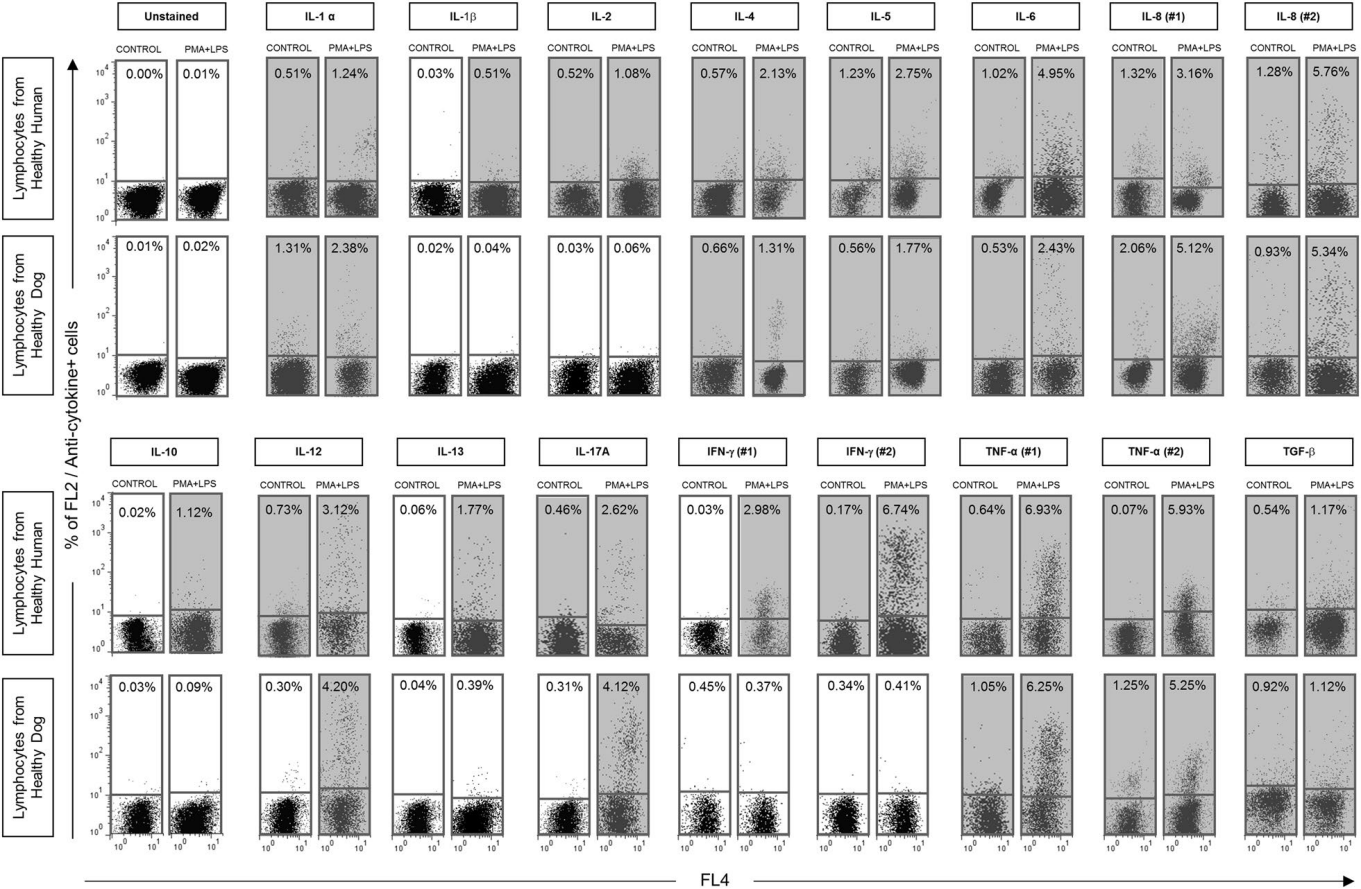
Cross-reactivity of a range of anti-human cytokine mAbs with canine cytokines following short-term whole blood cultures in vitro. *Dot plot* distribution graphs of empty channel (FL4 fluorescence) versus PE-emission channel (FL2) were used to quantify the percentage of cytokine^+^ lymphocytes in unstimulated controls (*left panels*) and PMA + LPS stimulated cultures (*right panels*). Only Q1 and Q4 quadrants are represented. *Gray background* highlights the reactivity above 0.5 % in the quadrant Q1

**Fig. 3:**
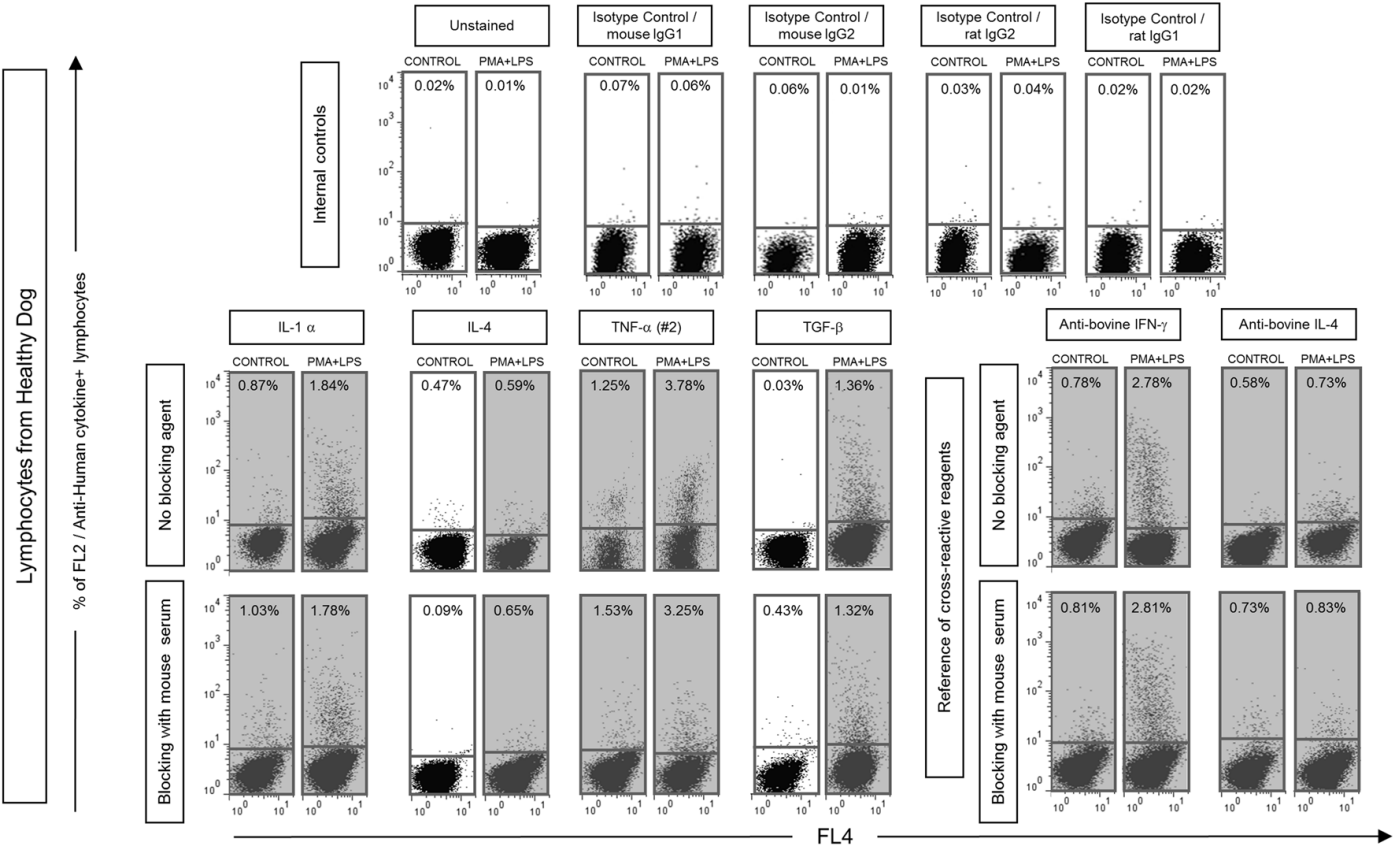
Internal controls to validate the cross-reactivity of anti-human cytokine mAbs with canine cytokines following short-term whole blood cultures in vitro. Unspecific binding were monitored by using isotypic matching reagents (*top panels*). Blocking strategy with host serum were also applied (*bottom panels*). Controls of reference cross-reactive mAbs (anti-bovina IFN-γ and IL-4) were also included. *Dot plot* distribution graphs of empty channel (FL4 fluorescence) versus PE-emission channel (FL2) were used to quantify the percentage of cytokine^+^ lymphocytes in unstimulated controls (*left panels*) and PMA + LPS stimulated cultures (*right panels*). Only Q1 and Q4 quadrants are represented. *Gray background* highlights the reactivity above 0.5 % in the quadrant Q1

**Fig. 4:**
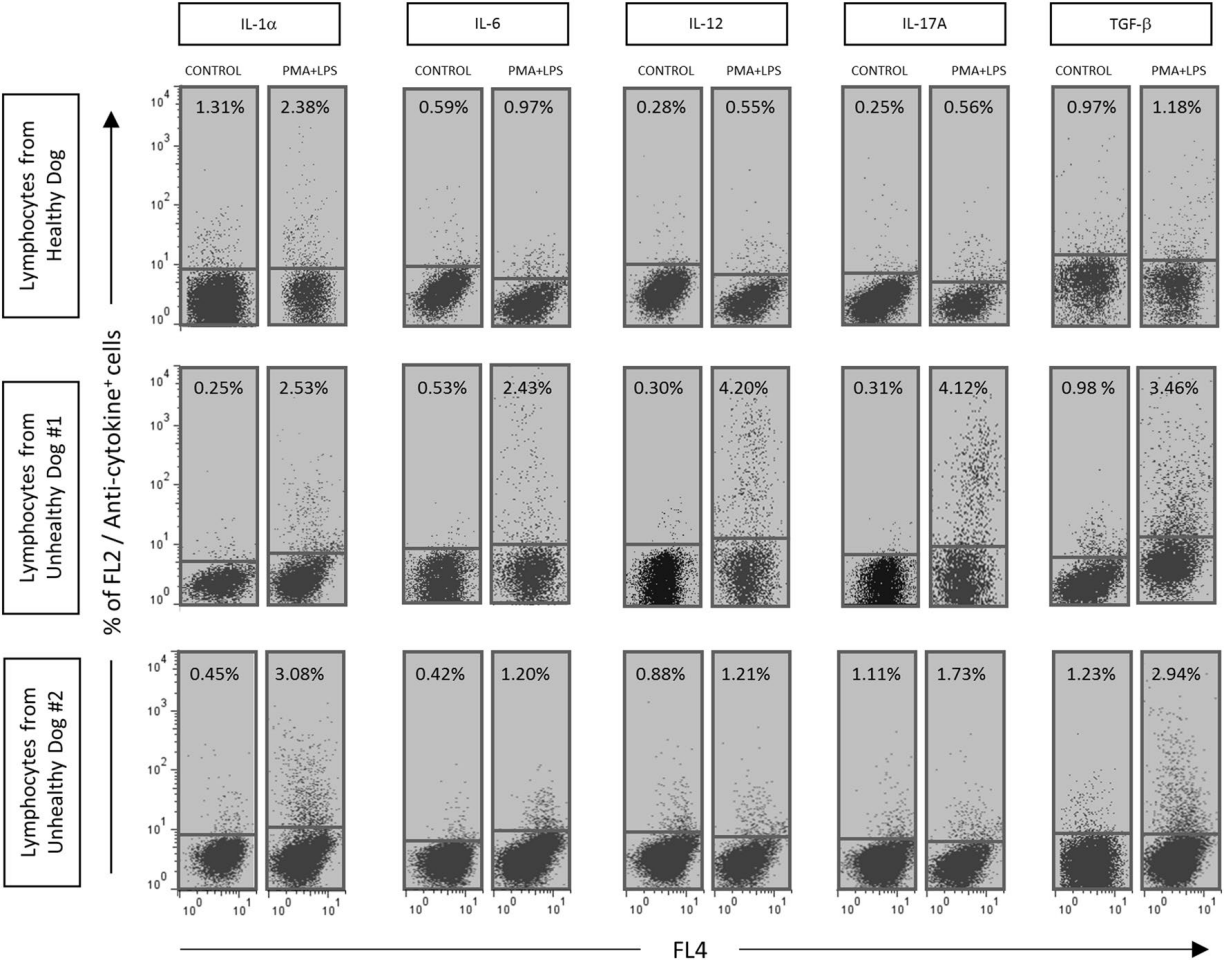
Applicability of cross-reacted anti-human cytokine mAbs (IL-1-α, IL-6, IL-12, IL-17A and TGF-β) to quantify cytokine^+^ lymphocytes from unhealthy dogs, including peritonitis (Dog #1) and dermatitis (Dog #2). *Dot plot* distribution graphs of empty channel (FL4 fluorescence) versus PE-emission channel (FL2) were used to quantify the percentage of cytokine^+^ lymphocytes in unstimulated controls (*left panels*) and PMA + LPS stimulated cultures (*right panels*). Only Q1 and Q4 quadrants are represented. *Gray background* highlights the reactivity above 0.5 % in the quadrant Q1

**Fig. 5:**
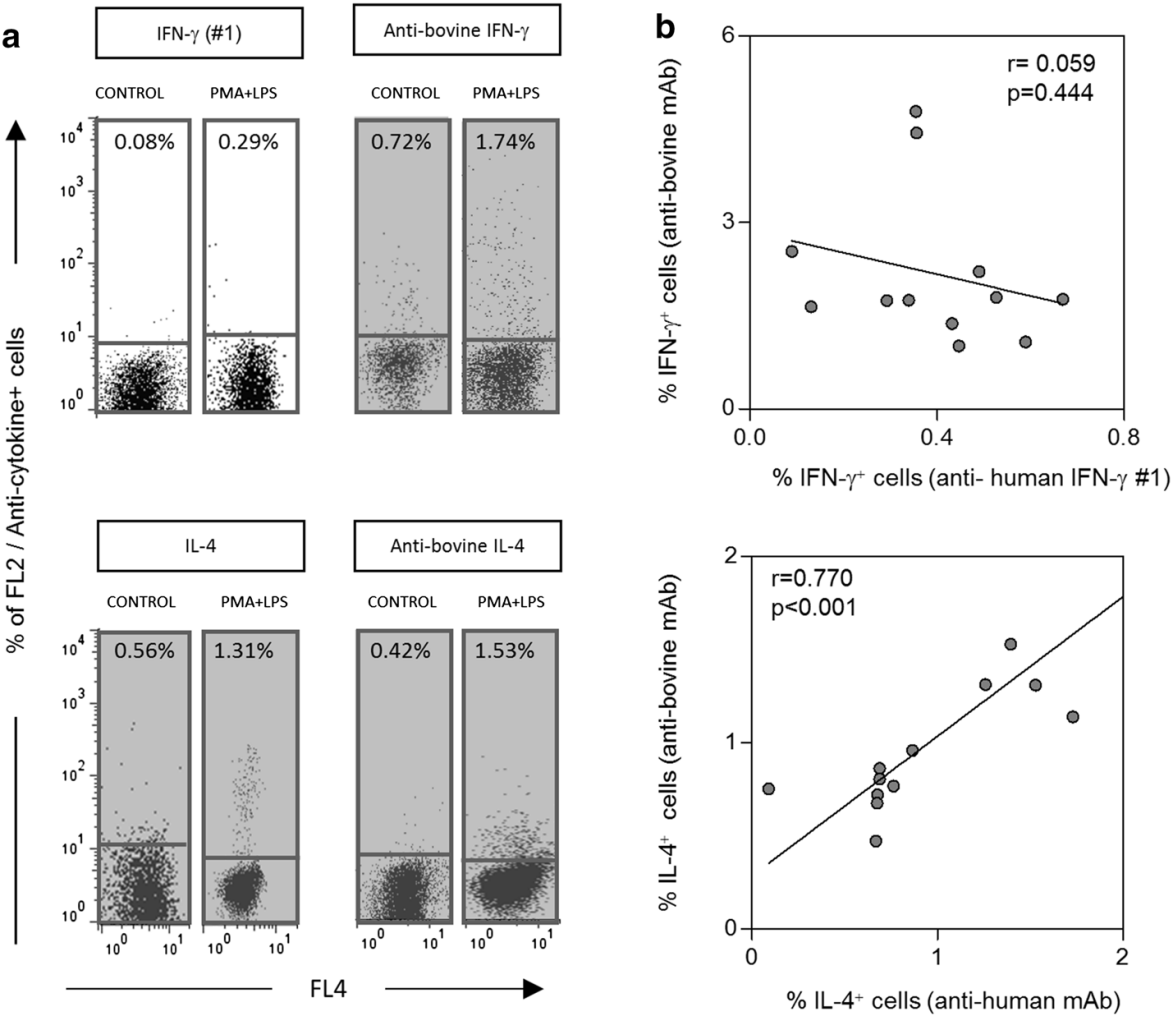
Correlation analysis of cytokine^+^ lymphocytes detected by commercially available anti-human cytokine mAbs (IL-4 and IFN-γ) and the reference cross-reactivity reagent (anti-bonive IL-4 and IFN-γ). **a** *Dot plot* distribution graphs of empty channel (FL4 fluorescence) versus PE-emission channel (FL2) were used to quantify the percentage of cytokine^+^ lymphocytes (IFN-γ #1—*upper panel* and IL-4—*bottom panel*). Only Q1 and Q4 quadrants are represented. *Gray background* highlights the reactivity above 0.5 % in the quadrant Q1. **b** Spearman correlation plots of the frequency of cytokine^+^ lymphocytes stained with each mAbs (anti-human versus reference anti-bovine mAbs)

Flow cytometry acquisition and analysis were performed in a FACScalibur™ flow cytometer (Becton–Dickinson), interfaced to an Apple G3 FACStation using the Cell Quest software (Franklin Lakes, NJ, USA). After acquiring 20,000 events/tube, the data were used to analyze the different cytokine-expressing lymphocytes. The FlowJo 7.6.1 (Tree Star, Ashland, OR, USA) software was used for data analysis. Figure 1 summarizes the data analysis used to quantify the frequency of lymphocytes expressing intracytoplasmic cytokines. The lymphocyte and monocytes gating was based on their size (forward scatter—FSC) and granularity (side scatter—SSC). Those are morphometric features on dot plot distributions, where they are confined into a region of low size and complexity (R1) (Fig. 1a). Cytokine-expressing lymphocytes were quantified using Fluorescence FL4 versus Fluorescence FL2/anti-cytokine-PE dot plots. Then, the FL2 positive quadrant (Q1) was selected to show the cytokine production (Fig. 1b). When considering antibodies giving weak staining signals, the most likely explanation is that the fluorescent signals in some cases simply were too weak to fall within our criteria of being regarded as positive (more than 0.5 % of the lymphocytes positive from stimulated culture). The results were expressed as percentage of lymphocytes that express the cytokine of interest (Fig. 1c). This pattern (Fig. 1c) was used subsequently in all experiments (Figs. 2, 3, 4, 5).

### Confocal analysis of intracellular cytokines

After the short-term whole culture, 200 μl of cell suspension were used for immunostaining for confocal microscopy. In 5 ml polypropylene tubes (Becton–Dickinson), cell suspension was incubated with 5 μl anti-human CD3 fluorescein isothiocyanate (FITC) labeled mAb (Serotec, Kidlington, UK) for 30 min at room temperature in the dark. Then, the erythrocytes were lysed by adding 4 ml of FACS Lysing Solution (Becton–Dickinson) under vortex mixing. After 5 min, cells were washed by centrifugation (600×*g*, 7 min, room temperature). Then, samples were incubated with FACS Permeabilizing Solution (0.015 M phosphate buffered saline—PBS, supplemented with 0.5 % bovine serum albumin, 0.5 % of saponin, and 0.1 % sodium azide—Becton–Dickinson, San Jose, CA, USA) for 5 min and washed by centrifugation (600×*g*, 7 min, room temperature). Following, the cells were resuspended in 200 μl of FACS Permeabilizing Solution and 50 μl of the suspension were immunostained by adding 1 μl with PE-labeled anti-cytokine mAbs [IL-4, IL-8(#2) and TGF-β]. Cells were incubated in the dark for 30 min at room temperature. Then, the cells were washed twice with 2 ml of FACS buffer, resuspended and fixed with 50 μl of FACS FIX Solution (BD Pharmigen). This cell suspension was placed in the CytoSpin apparatus (Cytospin II, Shandon) and centrifuged (500×*g*, 10 min). The samples were mounted and covered by glass slides using the antifade agent mowiol (Polysciences, Inc., Warrington, PA, USA). The material was visualized using a Zeiss laser scanning inverted microscope (Axiovert LSM510) (Carl Zeiss MicroImaging, Inc., Thornwood, NY, USA). The Zeiss LSM Image Browser (Version 4.2.0.121) software was used for image analysis.

## Results

### Anti-human cytokine mAbs cross-reactivity against canine cytokines

The analysis of the frequencies of lymphocytes expressing intracytoplasmic cytokines were determined by the strategy of conventional analysis as described using TNF-α as an example (Fig. 1). After stimulation, an increase (7.25 %) in the percentage of cells expressing the cytokine was observed as compared with control culture (1.25 %) (Fig. 1, lower right). Following this pattern, all subsequent data were obtained for the other cytokines and percentages above 0.5 % were considered reactive, highlighted by gray panels in all flow cytometric representative figures. Using a panel of seventeen anti-cytokine mAbs it was possible to detect cross-reactivity between anti-human cytokine mAbs with canine intracytoplasmic cytokines for eleven cytokines [IL-1α, IL-4, IL-5, IL-6, IL-8 (#1 and #2), IL-12, IL-17A, TNF-α (#1 and #2) and TGF-β]. As expected, higher production of some cytokines such as IL-8, IL-12, IL-17A and TNF-α was observed in the PMA + LPS stimulated cultures as compared with control cultures. On the other hand, anti-IFN-γ mAbs (#1 and #2) were very specific in detecting those cytokines in the stimulated cultures from human lymphocytes and were not able to recognize canine IFN-γ. A similar profile was found for the anti-human IL-2 mAb tested. Finally, low levels of IL-1β and IL-10 was observed in human lymphocytes and was absent in canine cells (Fig. 2).

**Fig. 6:**
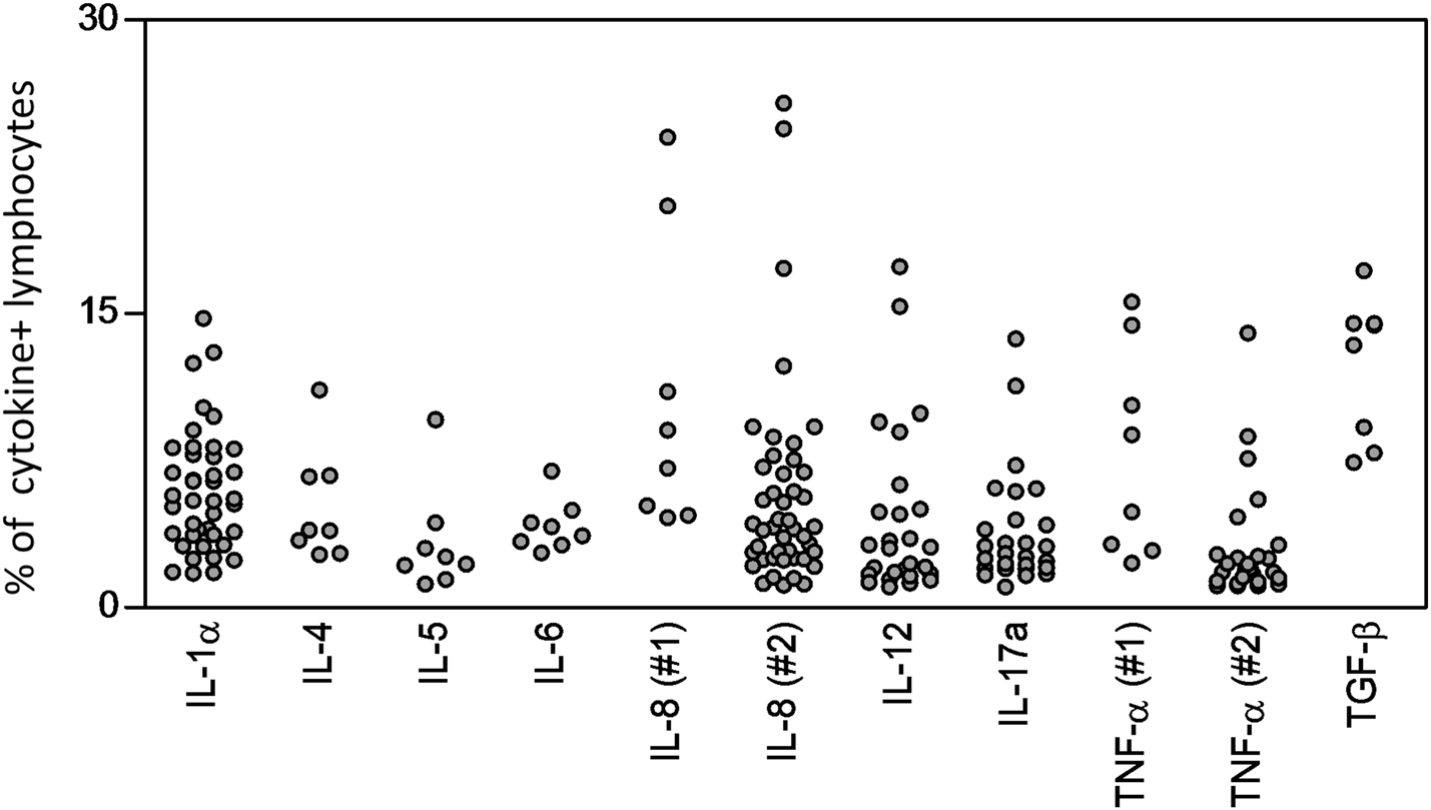
Range of intracytoplasmic cytokine labeling in peripheral blood lymphocytes from healthy dogs detected by the selected cross-reactivity reagents. The frequency of cytokine^+^ lymphocytes was assessed in a total of 8-45 healthy dogs. The results are expressed as scattering of individual values for IL-1α, IL-4, IL-5, IL-6, IL-8 (#1), IL-8 (#2), IL-12, IL-17A, TNF-α (#1), TNF-α (#2) and TGF-β

The range of intracytoplasmic cytokine labeling in peripheral blood lymphocytes from healthy dogs detected by the selected cross-reactivity reagents are shown in Fig. 6.

### Isotype matching controls and Fc-γ-R blocking

To address whether the cross-reactivity of the anti-human cytokine mAbs with canine cytokines could be a non-specific reaction via Fcγ-R binding, an experiment including internal isotypic controls as well as blocking pre-incubation in the presence of 2 % of mAbs host sera reagent was performed (Fig. 3). This strategy was used to provide a major source of unlabeled IgG for species-specific Fcγ-R blocking by competitive binding. Moreover, a parallel analysis of intracytoplasmic staining with the reference cross-reactive reagents was also performed. Consistent with our previous observations (Fig. 2), no differences could be observed in the “presence” of “Fc-γR blocking for the anti-human IL1-α, IL-4, TNF-α (#2) mAbs and anti-bovine IL-4 and IFN-γ control mAbs (Fig. 3).

### Applicability of anti-human cytokine mAbs to characterize the immunological status of diseased dogs

After the cross-reactivity panel was established, we performed an additional experiment to evaluate the applicability of the selected anti-human cytokine mAbs to characterize the immunological status of diseased dogs including peritonitis (Dog #1) and dermatitis (Dog #2). Data analysis demonstrated that both dogs exhibited a different cytokine pattern depending on the pathology (Fig. 4). Dog #1 exhibited an increase in the intracytoplasmic levels of IL-6, IL-12, IL-17A and TGF-β, whereas dog #2 showed increased expression of IL-1α, IL-12, 17A and TGF-β by lymphocytes.

Two commercially available anti-bovine cytokine mAbs (IL-4 and IFN-γ) are currently used to mark canine leukocytes producing theses cytokines. Theses mAbs were used to compare the reactivity observed by anti-human cytokine-cross-reacting mAbs tested. As expected, a positive strong correlation between the percentage of positive cells marked with anti-human and anti-bovine IL-4 mAbs was observed (r = 0.770 and *P* < 0.001), while no correlation was observed between the anti-human IFN-γ mAb (not reactive) and anti-bovine IFN-γ mAb (r = 0.059 and *P* = 0.444) (Fig. 5).

**Fig. 7:**
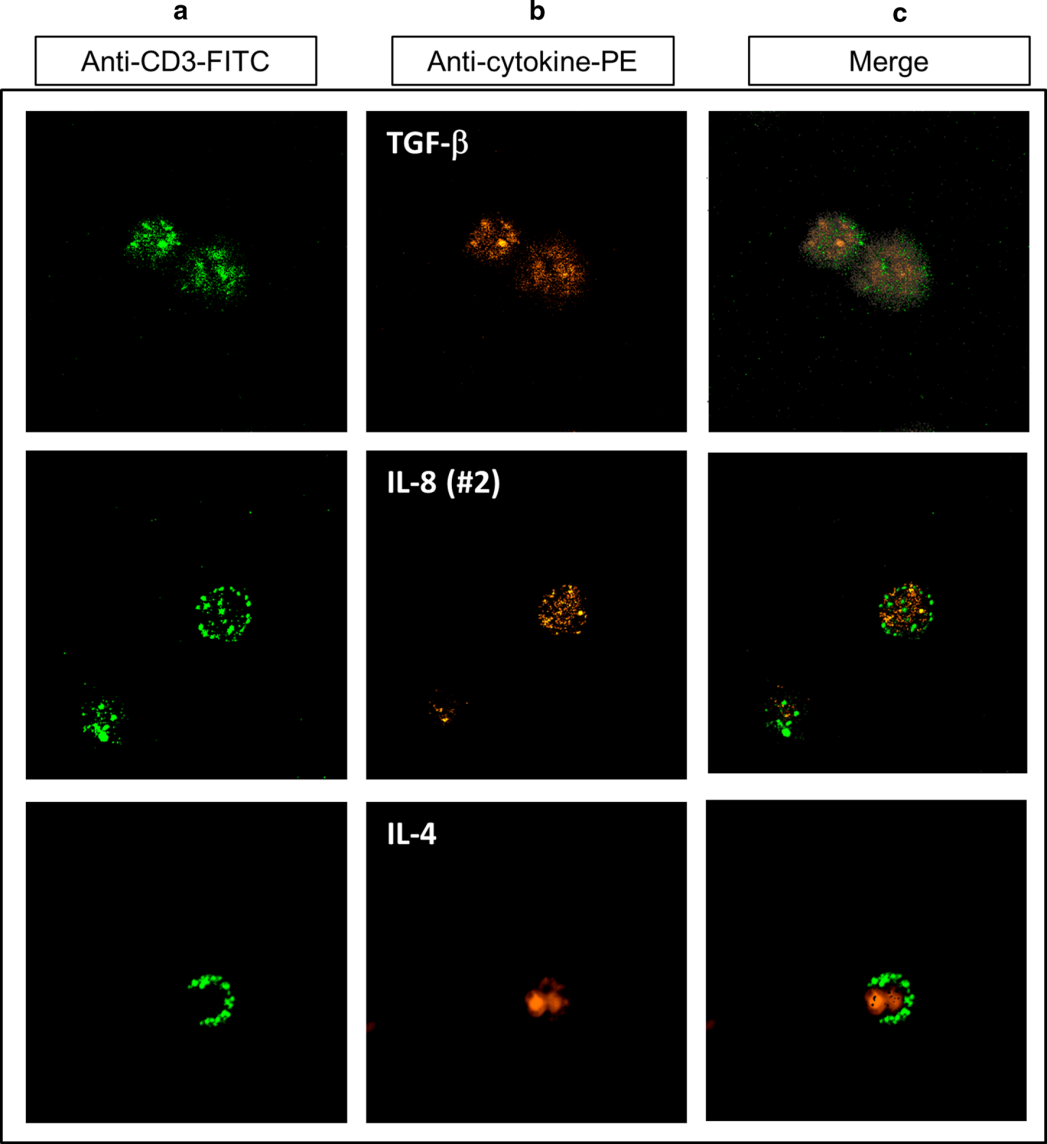
Confocal analysis of intracytoplamic stained cytokines. **a** Stimulated cells (PMA + LPS) were stained with FITC-labeled rat anti-canine CD3 and **b** PE-labeled anti-human TGF-β, IL-8 (#2) and IL-4. Images from both staining were merged (**c**)

In order to confirm the mAbs cross-reactivity in situ, canine T-lymphocytes were stained with anti-dog CD3-FITC plus anti-human cytokines-PE [TGF-β, IL-8 (#2) and IL-4]. The cells were evaluated employing Zeiss laser scanning inverted microscope. Cellular co-localization of CD3 and TGF-β, IL-8 (#2) or IL-4 was detected (Fig. 7). These results confirmed the cross-reactivity data shown by the flow cytometric analysis.

## Discussion

In this work we have screened 17 monoclonal antibodies against human cytokines and their cross-reactivity against canine lymphocytes. Cross-reactivity studies using well-defined mAbs are an important and rapid tool to obtain reagents that will be suitable for immunological studies in veterinary medicine.

The rationale of performing such studies lies in the fact that it is much more economical to identify useful immunological cross-reactivity of existing well-defined monoclonal antibodies than to produce new reagents, since the development and production of new mAbs is time-consuming and expensive. In some species a limited number of marker antibodies are available. Therefore, the search for monoclonal antibodies generated against antigens derived from other species for their cross-reactivity and systematic studies have been performed [18, 20, 25–28]. Some cross-reacting antibodies have been employed to study canine hematopoietic cells [29, 30], canine lymphomas [31], canine lymphocyte subsets [32] or the canine leukocyte adhesion deficiency syndrome (CLAD) [33].

The advent of intracellular cytokine assessment by flow cytometry, through staining of permeabilized cells with fluorochrome conjugated anti-cytokine antibodies, permits large numbers of cells of known phenotypes to be examined. Flow cytometry allows both quantitative and statistical analysis of variable cell molecules expressed on the cellular membrane or cytoplasm in each individual cell within a short time period. Intracellular cytokine staining is quite important in defining functional subsets in mixed T cell populations. In this work, we employed a standard protocol for in vitro short-term whole blood culture using flow cytometry-based immunofluorescence assay to test the cross-reactivity of seventeen commercially available PE-labeled anti-human cytokine mAbs. Basal cytokine and PMA + IONO + LPS-induced plus cytokine profiles were evaluated and the cross-reactivity with canine intracytoplasmatic cytokines assessed by comparative analysis with the standard reactivity in human lymphocytes. The results of this study showed that short culture times (4 h) for canine whole-blood samples are satisfactory for the cytokine evaluation. Data analysis demonstrated that eleven out of seventeen anti-human cytokine mAbs (IL-1α, IL-4, IL-5, IL-6, IL-8 (#1 and #2), IL-12, IL-17A, TNF-α (#1 and #2), TGF-β) cross-reacted with canine cytokines. Otherwise, no cross-reactivity with the canine cytokines was observed by six anti-human cytokine mAbs [IL-1β, IL-2, IL-10, IL-13, IFN-γ (#1 and #2)].

Comparison of the available amino acid sequences of the human cytokines for which the mAbs were generated and canine cytokines showed homologies from 49 % up to 96 % (Table 2) (http://www.ncbi.nlm.nih.gov/BLAST/) consistent with an occurrence of cross-reactivity. Maintenance of epitopes on molecules may reflect functional importance of the region of the molecule recognized, and an evolutionary pressure to conserve those regions [34]. Some results found in this study and by others [11, 22, 23] do not completely support the idea that high homology lead to reactivity, probably because the epitopes recognized by mAbs are not in regions that have been conserved evolutionarily. Comparative studies of the reactivity with cells from several species have shown that each antibody recognizes a different epitope [12]. Dernfalk et al. [11] tested five commercially available antibodies against human TNF-α towards ovine and bovine, but found only two antibodies with satisfactory reactivity with the ovine, but none with the bovine TNF-α. Thus, it is often necessary that several antibodies against the same antigen should be tested, which could suggest that a cross-reacting mAb could be found if other different clones were screened.

**Table 2:** Homology of the amino acid sequences between the canine and human or bovine cytokines targeted by the evaluated anti-human or anti-bovine mAbs

Species	Cytokines	Homology to canines (%)
Human	IL-1-α	70
	IL-1-β	78
	IL-2	74
	IL-4	49
	IL-5	65
	IL-6	59
	IL-8	77
	IL-10	78
	IL-12	84
	IL-13	72
	IL-17A	81
	IFN-γ	85
	TNF-α	91
	TGF-β	96
Bovine	IL-4	65
	IFN-γ	76

Anti-human IL-8, IL-12, IL-17A, and TNF-α (#1, #2) mAbs strongly reacted against intracellular cytokines in the canine lymphocytes. These results are supported by a high degree of similarity found among human and canine cytokines evaluated (Table 2). They showed high homology among species (77, 84, 81, 85 and 91 %, respectively). In spite of the lower homology between human and canine IL-6 (59 %), the monoclonal antibody anti- human IL-6 showed cross-reactivity with the canine cytokine. On the other hand, IL-1β, IL-2, IL-10 and IL-13 presented high homology between canine and human (78, 74, 78, 72 %, respectively), but no cross-reactivity was observed by the anti-human mAb clones tested against those cytokines.

IL-8 is a very important pro-inflammatory cytokine, the possibility to study its production by canine leukocytes through flow cytometry employing mAbs could help to understand the immunopathology in different diseases (infectious or not). In addition, cross-reactivity of anti-human IL-17A mAb opens a new possibility for investigating the relevance of TH17 cells in the immune response of dogs. Although presenting homology of 78 %, the human anti-IL-10 mAb did not react with the canine cytokine. The finding of a mAb reactive against canine IL-10 would be very useful, since this is an important cytokine involved in immunoregulation. It may induce down-regulation of Th1 cytokines expression, MHC class II antigens, and acting as co-stimulatory molecule on macrophages. It also enhances B cell survival, proliferation, and antibody production. IL-10 can block NF-κB activity, and is involved in the regulation of the JAK-STAT signaling pathway [35, 36].

Results for both human anti-TNF-α mAb (Clone B-C7 or mab11) showed a good cross-reactivity with canine TNF-α, similar to the pattern found for human cells. These results are not surprising because this cytokine has great homology between human and canine. This finding is promissory, since that TNF-α is a important innate pro-inflammatory cytokine involved in protection against intracellular pathogens, and sometimes the identification of cross-reactivity among evolutionarily related species have been difficult [11, 22].

To validate the cross-reactivity findings, an additional experiment was performed to evaluate the applicability of the selected anti-human cytokine mAbs. It was performed to characterize the immunological status of dogs under pathological conditions including peritonitis (Dog #1) and dermatitis (Dog #2). We observed that both dogs exhibited a different cytokine patterns depending on the pathology. The dog with peritonitis exhibited increased intracytoplasmic levels of IL-6, IL-12, IL-17A and TGF-β, whereas the dog with dermatitis presented increased expressions of IL-1α, IL-12 and TGF-β in lymphocytes. Increased expression for most cytokines in cells from dogs with peritonitis (Dog #1) and dermatitis (Dog #2) as compared with healthy animals, reinforced the importance and applicability of the cross-reactive mAbs to increase the portfolio of mAbs against canine cytokines available.

The results presented here demonstrate the potential of cross-reactive monoclonal antibody against distinct species by immunofluorescence assays to monitor the frequency of cells producing cytokines. Additonately, this approach may further permit the evaluation of immune response in various canine disorders with the potential for monitoring therapy and vaccine protocols.

## Conclusion

This study described a wide range of anti-human cytokine mAbs (IL-1α, IL-4, IL-5, IL-6, IL-8(#1, #2), IL-12, IL-17A, TNF-α (#1, #2), TGF-β) that cross-reacted with canine cytokines and could be useful canine immunological biomarkers for pathological conditions employing flow cytometry and fluorescence microscopy.
